# Obesity and Eye Diseases

**DOI:** 10.3390/nu18162692

**Published:** 2026-08-18

**Authors:** Kamila Pieńczykowska, Anna Bryl, Małgorzata Mrugacz

**Affiliations:** 1 Doctoral School, Medical University of Bialystok, ul. Jana Kilińskiego 1, 15-089 Bialystok, Poland; mynameiskama@gmail.com; 2 Department of Pediatric Ophthalmology and Strabismus, Medical University of Bialystok, ul. Jana Kilińskiego 1, 15-089 Bialystok, Poland; anna.bryl@umb.edu.pl

**Keywords:** obesity, dry eye, glaucoma, cataracts, age-related macular degeneration, diabetic retinopathy, myopia

## Abstract

**Background:** Obesity has become a major global public health challenge, affecting individuals across all age groups and contributing to a wide range of systemic disorders. Beyond its well-established associations with cardiovascular and metabolic diseases, obesity is increasingly recognized as a chronic inflammatory condition that influences the structure and function of multiple organs, including the eye. **Methods:** A comprehensive literature review was conducted between November 2025 and June 2026 using PubMed, Web of Science, and Google Scholar. Priority was given to recent studies evaluating the impact of obesity and obesity-related metabolic disturbances on ocular health. **Results:** Available evidence indicates that obesity is associated with an increased risk of several ophthalmic conditions, including age-related macular degeneration, diabetic retinopathy, glaucoma, cataracts, and retinal vein occlusion. Multiple anthropometric measures of adiposity have been positively associated with diabetic retinopathy risk. Although findings regarding glaucoma remain heterogeneous, metabolic syndrome and its components, particularly impaired glucose metabolism, appear to increase glaucoma susceptibility. Obesity has also been linked to dry eye disease through inflammatory and tear film alterations. **Conclusions:** Obesity exerts significant effects on ocular health through complex metabolic, inflammatory, and vascular pathways. Recognition of obesity as a modifiable risk factor for eye disease may facilitate earlier detection, targeted prevention strategies, and improved multidisciplinary management. Further longitudinal and mechanistic studies are needed to clarify causal relationships and determine whether effective obesity treatment can reduce the burden of vision-threatening ocular disorders.

## 1. Introduction

Obesity has emerged as one of the most important global health challenges of the 21st century, with increasing prevalence across both developed and developing regions. In 2014, obesity was more prevalent than underweight among men in 136 of 200 countries and among women in 165 countries [1]. More recent data indicate that obesity remains highly prevalent among adults and is also increasing among children and adolescents [2,3,4]. These trends are clinically important because obesity is associated not only with cardiovascular and metabolic disorders but also with chronic systemic inflammation, hormonal dysregulation, insulin resistance, and altered lipid metabolism.

**Figure 1:**
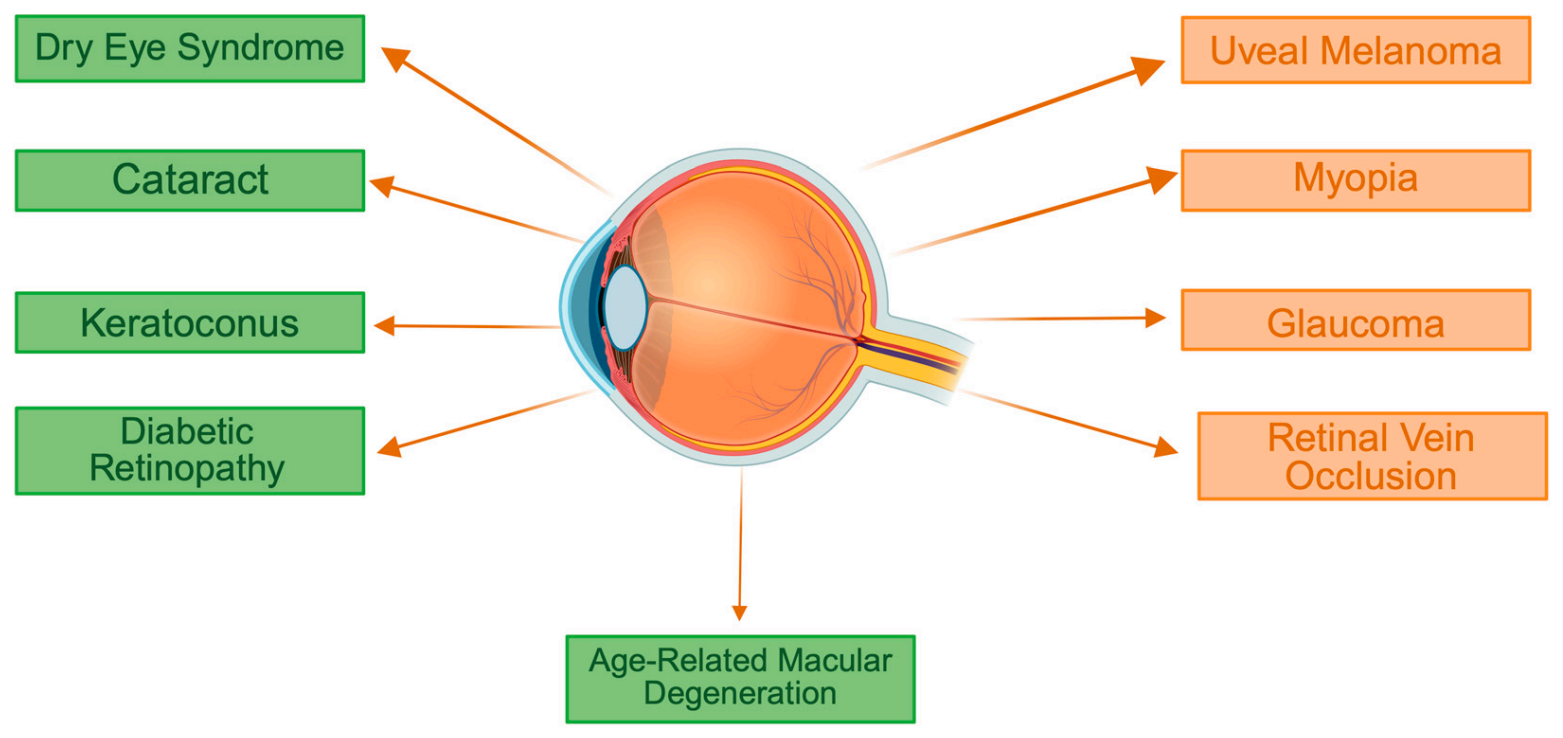
Presentation of eye diseases related to obesity. Green color boxes mean that there is consistent evidence that obesity increases the risk of these diseases and orange color means that the evidence is complex. Created in BioRender. Pieńczykowska, K. (2026) https://BioRender.com/qzqbjzl (accessed on 14 August 2026).

Obesity is characterized by excessive accumulation of adipose tissue, but BMI alone does not fully capture the distribution or metabolic consequences of adiposity. BMI may overestimate adiposity in individuals with high lean mass and underestimate body fat in some individuals near conventional obesity thresholds. Consequently, complementary measures such as waist circumference, waist-to-hip ratio, waist-to-height ratio, body fat percentage, and direct assessments of body composition may provide additional information regarding metabolic risk [5,6]. This distinction is particularly relevant to ophthalmic research because different measures of adiposity may demonstrate different relationships with ocular outcomes, as shown in Figure 1.

Adipose tissue is metabolically active and participates in endocrine, immune, and inflammatory signaling. In obesity, adipocyte hypertrophy and impaired adipose tissue function are accompanied by increased production of inflammatory mediators, including tumor necrosis factor-alpha (TNF-α), interleukin-6 (IL-6), and interleukin-1 beta (IL-1β) [7,8].

The relationship between obesity and ocular disease, however, is not uniform. Epidemiological studies have reported positive, neutral, and occasionally inverse associations depending on the ocular outcome and the population studied. For example, central adiposity may be more strongly associated with some ocular diseases than BMI, while obesity-related metabolic dysfunction may be more informative than body weight alone [9]. Differences in age, sex, ethnicity, diabetes, hypertension, dyslipidemia, lifestyle, physical activity, and other comorbidities may further modify these relationships. Excess lipid availability may also promote ectopic lipid accumulation, mitochondrial dysfunction, reactive oxygen species (ROS) production, endoplasmic reticulum stress, and impaired insulin signaling [10,11,12,13]. These systemic alterations may affect tissues with high metabolic demands, including the retina, retinal pigment epithelium (RPE), optic nerve, and ocular surface.

The aim of this narrative review is therefore not only to summarize reported associations between obesity and ocular diseases but also to critically examine their consistency, potential confounding factors, biological plausibility, and clinical relevance. Particular attention is given to the distinction between epidemiological, genetic, experimental, and interventional evidence, as well as to nutritional factors that may influence obesity-related ocular pathology.

## 2. Materials and Methods

This narrative review was conducted between November 2025 and June 2026 to synthesize and critically evaluate the available evidence concerning systemic obesity, metabolic dysfunction, nutritional factors, and ocular disease.

A comprehensive literature search was performed using PubMed, Web of Science, and Google Scholar. Search terms included combinations of obesity-related concepts, such as “obesity,” “overweight,” “body mass index,” “BMI,” “adiposity,” “central obesity,” “visceral adiposity,” “waist circumference,” “waist-to-hip ratio,” “metabolic syndrome,” “insulin resistance,” “inflammation,” “diet,” and “nutrition,” with ocular disease terms including “dry eye disease,” “keratoconus,” “myopia,” “cataract,” “age-related macular degeneration,” “glaucoma,” “diabetic retinopathy,” “retinal vein occlusion,” and “uveal melanoma”.

The search prioritized publications from approximately the previous 10–15 years, while older studies were retained when they represented seminal epidemiological observations or provided relevant mechanistic evidence.

Peer-reviewed original studies, systematic reviews, meta-analyses, cohort studies, case–control and cross-sectional studies, Mendelian randomization (MR) analyses, clinical and interventional studies, and relevant animal and experimental studies were considered. Studies were eligible when they investigated obesity, adiposity, obesity-related metabolic factors, or nutritional exposures in relation to ocular disease, ocular structure or function, or biological pathways relevant to ocular pathology.

Non-peer-reviewed publications, studies not available in English, and publications without relevant primary or synthesized data were excluded.

**Figure 2:**
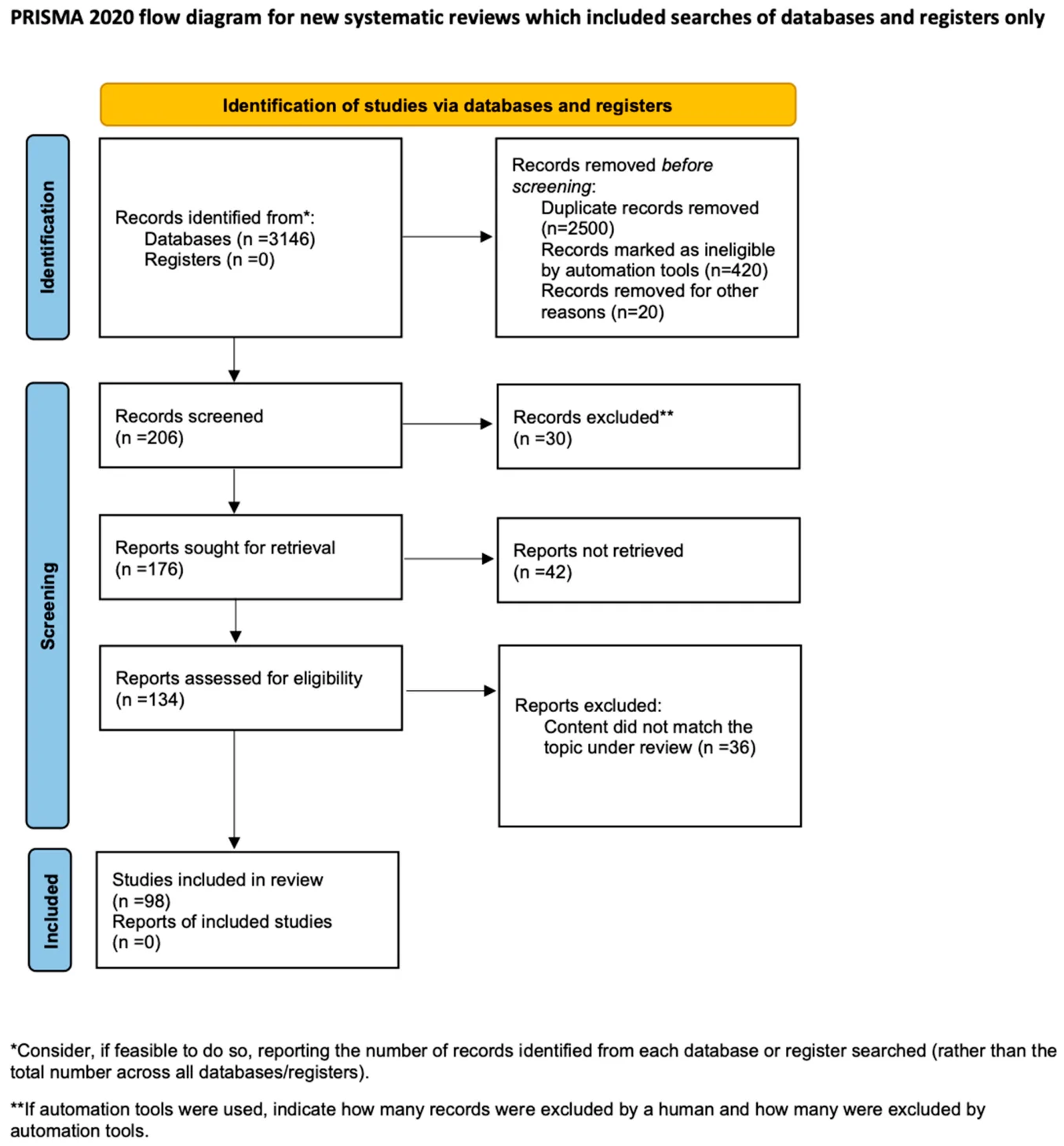
PRISMA flowchart of study selection process.

Study selection consisted of an initial screening of titles and abstracts followed by full-text assessment of potentially relevant articles. Reference lists of relevant reviews and primary studies were also examined to identify additional publications. Ultimately, 98 studies were included in the final narrative synthesis. A literature selection flowchart is presented in Figure 2.

Relevant information was organized according to ocular disease and included the study population, study design, obesity or adiposity measure, ocular outcome, major findings, quantitative effect estimates where available, potential confounders, and proposed biological mechanisms.

Because this was a narrative review, no formal quality assessment or risk-of-bias evaluation and no meta-analysis were performed. Consequently, the overall strength of the evidence should be interpreted cautiously because the included studies differ in design, population, methodology, sample size, and susceptibility to bias.

The evidence was interpreted according to study design. Observational studies were considered primarily as evidence of association and were not interpreted as demonstrating causality. MR studies were considered complementary evidence regarding potential causal relationships. Experimental animal and cellular studies were interpreted as mechanistic evidence and were not considered equivalent to human epidemiological findings. Clinical and interventional studies were considered separately because they may provide information regarding the modifiability of obesity-related ocular changes.

## 3. Overnutrition and Obesity

Overnutrition results from a sustained imbalance between energy intake and expenditure and promotes progressive expansion of adipose tissue. Initially, adipose tissue accommodates excess energy through adipocyte hypertrophy and hyperplasia. With persistent energy surplus, however, adipose storage capacity may become insufficient, promoting ectopic lipid deposition and accumulation of free fatty acids and potentially toxic lipid intermediates [10,11].

Lipotoxicity may impair mitochondrial, endoplasmic reticulum, and lysosomal function, resulting in increased ROS production and activation of inflammatory signaling pathways. Persistent inflammation may subsequently interfere with insulin signaling and glucose homeostasis, contributing to systemic metabolic dysfunction [11,12]. Leptin concentrations are also increased in obesity, while resistance to leptin-mediated appetite regulation may develop [13].

Importantly, obesity should therefore be considered a heterogeneous metabolic state rather than simply an increase in body weight. Individuals with similar BMI values may differ substantially in visceral adiposity, insulin sensitivity, lipid profile, inflammatory activity, and cardiometabolic health. This heterogeneity may partly explain why studies using BMI, waist circumference, body fat percentage, or visceral adiposity indices report different associations with ocular outcomes.

### 3.1. Common Molecular and Metabolic Mechanisms Linking Obesity to Ocular Disease

Although individual ocular diseases have distinct pathogenic pathways, several biological mechanisms recur across multiple conditions.

#### 3.1.1. Chronic Low-Grade Inflammation

Obesity is associated with persistent low-grade systemic inflammation and increased circulating concentrations of mediators such as TNF-α, IL-6, and IL-1β [8]. These mediators may influence ocular tissues directly or contribute to endothelial dysfunction, oxidative stress, immune-cell activation, and disruption of tissue homeostasis.

Inflammatory signaling may therefore represent a common pathway connecting obesity with ocular surface disease, retinal degeneration, optic nerve injury, and retinal vascular disease. However, the presence of systemic inflammation does not establish that obesity is a direct cause of any particular ocular disease.

#### 3.1.2. Oxidative Stress and Mitochondrial Dysfunction

Excess nutrient availability and ectopic lipid accumulation can increase mitochondrial stress and ROS production [11,12]. Ocular tissues are particularly susceptible to oxidative damage because of their high oxygen consumption, intense metabolic activity, and exposure to light.

Experimental evidence indicates that obesity may increase retinal oxidative stress and complement activation [14], while high-fat dietary models have demonstrated structural and functional retinal abnormalities [15,16,17]. Similar processes may contribute to corneal and lens pathology.

#### 3.1.3. Insulin Resistance and Glucose Dysregulation

Insulin resistance is a central component of obesity-related metabolic dysfunction and may link obesity with ocular disease through hyperglycemia, advanced glycation end products, oxidative stress, and inflammatory signaling. This pathway is particularly relevant to cataract and diabetic retinopathy, but impaired glucose metabolism may also influence glaucoma risk [18,19].

Importantly, diabetes may act as a mediator or confounder in studies examining obesity and ocular disease. Consequently, associations attributed to obesity should be interpreted in the context of glycemic status.

#### 3.1.4. Dyslipidemia and Lipotoxicity

Altered triglyceride, cholesterol, and lipoprotein metabolism may influence ocular tissues directly or through vascular and inflammatory pathways. Dyslipidemia has been associated with meibomian gland dysfunction [20,21,22], while altered lipid metabolism may influence retinal carotenoid transport and macular health [23]. Lipid accumulation and metabolic dysfunction may also contribute to retinal vascular disease.

#### 3.1.5. Adipokines and Endocrine Signaling

Adipose tissue produces numerous signaling molecules, including leptin and other adipokines. Obesity-related alterations in adipokine signaling may influence inflammation, vascular function, oxidative stress, and tissue remodeling. ANGPTL7 represents one potentially important pathway in glaucoma because obesity has been associated with increased circulating and adipose tissue levels of ANGPTL7, while ANGPTL7 may influence aqueous humor outflow and intraocular pressure (IOP) [24,25].

#### 3.1.6. Gut Microbiome and Immune Dysregulation

Obesity-associated gut dysbiosis may impair intestinal barrier function and promote endotoxemia and systemic inflammation. Emerging evidence suggests that these changes may contribute to neuroinflammation and retinal or optic nerve injury [26]. This pathway remains biologically plausible but requires further investigation before its clinical significance in obesity-related ocular disease can be established.

**Figure 3:**
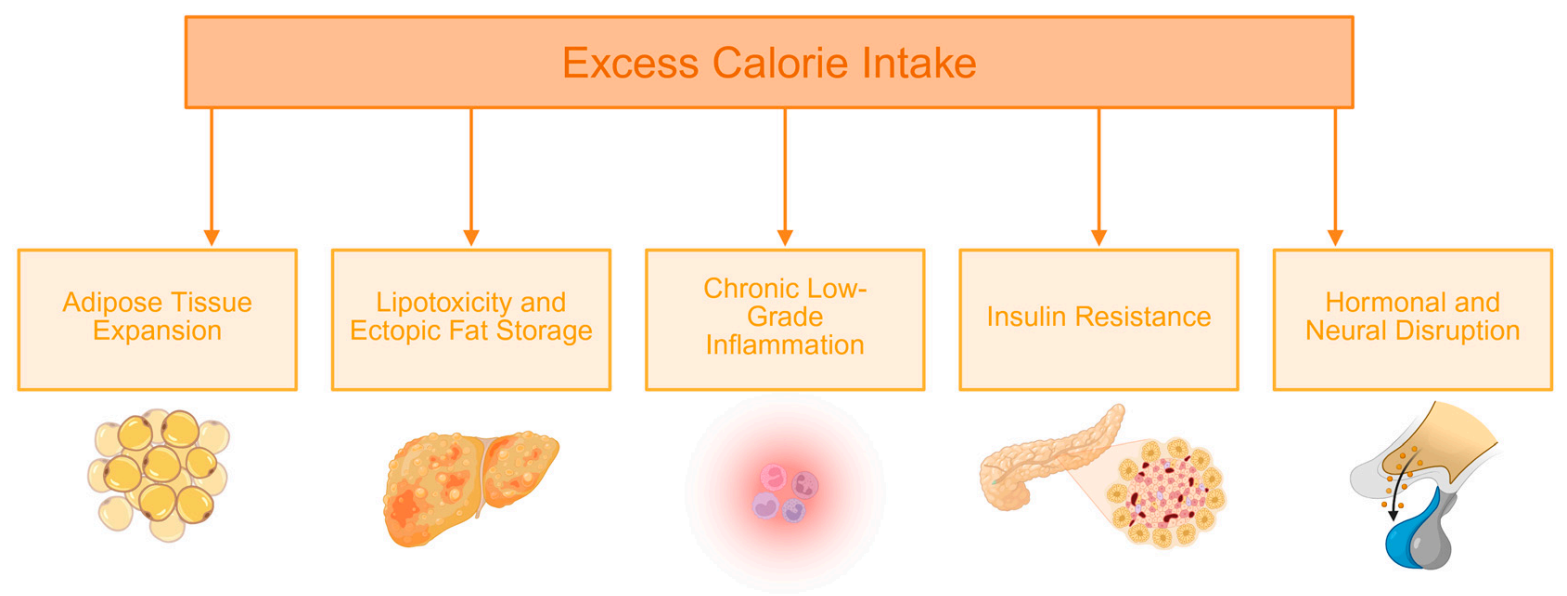
Presentation pathways leading from overnutrition to obesity. Created in BioRender. Pieńczykowska, K. (2026) https://BioRender.com/exmjdje (accessed on 13 July 2026).

Taken together, these mechanisms suggest that oxidative stress, mitochondrial dysfunction, inflammation, insulin resistance, lipid dysregulation, and immune alterations form an interconnected network rather than isolated pathways [Figure 3]. Their relative importance probably differs among ocular diseases [Table 1].

## 4. Dry Eye Syndrome

Individuals with obesity have a higher likelihood of developing dry eye syndrome [27]. Individuals with a very high BMI (≥30.0 kg/m^2^) had significantly higher odds of DES compared to those with a low BMI (<23.0 kg/m^2^) [28]. Obstructive sleep apnea was found to be an independent risk factor for increased prevalence of both dry eye disease and meibomian gland dysfunction [29]. Higher body mass index corresponds with a notable decrease in the presence of fern-like branches in the tear ferning test [30]. Compared with healthy children, those with obesity showed reduced tear meniscus area and height, shorter tear film breakup time, and lower Schirmer test scores. These measures were further decreased in children who had both obesity and insulin resistance [31]. HDL levels appear to play a significant role in tear film stability, while triglycerides and cholesterol affect the aqueous component of the tear film. These findings support the hypothesis that tear film deficiencies following gastric bypass surgery are predominantly evaporative rather than aqueous in nature [32]. Most meibum lipid species containing saturated fatty acids are elevated following a high-fat diet, along with signs of meibomian gland enlargement and increased tear production. This lipid imbalance is linked to changes in meibum composition, which is a central characteristic of meibomian gland dysfunction [20]. The meiboscore increased as weight, BMI percentile, and OSDI scores rose, and as tear film breakup time decreased. In addition, meibomian gland tortuosity worsened with increasing age and higher BMI percentile [21]. Advanced-stage meibomian gland dysfunction seems to be connected with serum triglyceride levels above 150 mg/dL and total cholesterol levels exceeding 200 mg/dL [22]. Trehalose may be beneficial in treating metabolic disorders such as diabetes and obesity by supporting glucose balance, improving how the body responds to insulin, and affecting fat metabolism [33]. Overall, the evidence supports a strong link between obesity, metabolic dysregulation, and deterioration of tear film and meibomian gland health.

## 5. Keratoconus

The relationship between obesity and keratoconus is supported by epidemiological and genetic evidence but remains insufficient to establish a definitive causal pathway. A Mendelian randomization study reported evidence consistent with a potential causal effect of BMI on keratoconus [34], while a large population-based study among adolescents also found an association between BMI and keratoconus [35]. Another genetic analysis reported evidence supporting general obesity as a potential causal factor but did not identify a comparable association for central obesity [36].

These findings are notable because they suggest that different aspects of adiposity may not have equivalent relationships with corneal disease. Experimental and clinical observations provide potential biological explanations. Obesity has been associated with corneal small-fiber nerve injury and increased dendritic-cell density [37]. Increased IL-8 expression has also been reported in keratoconic corneal epithelium [38], while oxidative stress and altered antioxidant responses are characteristic features of keratoconus [39].

A case report describing regression of keratoconus after gastric sleeve surgery provides an intriguing observation but cannot establish that weight reduction reverses the disease [40].

Overall, current evidence supports a possible relationship between obesity and keratoconus, with inflammation and oxidative stress representing plausible mechanisms. However, further prospective studies and mechanistic investigations are needed to determine whether adiposity independently influences corneal ectatic disease.

## 6. Myopia

The association between obesity and myopia appears to be strongest in children and adolescents, although findings are inconsistent across populations. Higher BMI, body fat percentage, and waist circumference have been associated with myopia in several studies [41,42,43,44]. An inverse L-shaped association has also been reported, suggesting that the relationship may not increase linearly across the full BMI range [45].

Sex and age may modify these associations. Some studies have reported stronger relationships in girls or males, whereas adult studies have produced less consistent results [42,46]. Genetic analyses have also suggested possible relationships between obesity-related traits and myopia, although the causal pathways may be complex [47].

Importantly, obesity should not be interpreted in isolation from environmental determinants of myopia. Outdoor activity is associated with a lower risk of myopia and may also influence the relationship between obesity and refractive development [48]. Near-work, educational exposure, sedentary behavior, and screen-based activities may also correlate with both adiposity and myopia risk. These shared lifestyle characteristics create the possibility of confounding or mediation.

Thus, although higher adiposity has been associated with myopia in several pediatric populations, current evidence does not support obesity as a primary or sufficient cause of myopia. Rather, adiposity appears to represent one component of a broader interaction involving genetic susceptibility, outdoor exposure, near-work, physical activity, sedentary behavior, and developmental factors.

## 7. Cataracts

The association between obesity and cataract has been examined using multiple measures of adiposity, including BMI, waist circumference, waist-to-hip ratio, and trunk fat percentage [49]. The consistency of these associations varies according to cataract subtype, sex, and metabolic status.

A dose–response meta-analysis reported that each 5 kg/m^2^ increase in BMI was associated with a 6% higher risk of age-related cataract and a 27% higher risk of posterior subcapsular cataract, whereas no significant association was observed for nuclear or cortical cataract in the overall analysis [50]. Other studies have demonstrated sex-specific relationships, including positive associations between general or abdominal obesity and cataract among women but inverse associations between abdominal obesity and some cataract subtypes among men [51].

Diabetes appears to be an important mediator or confounder. Studies examining multiple adiposity measures have suggested that diabetes partially mediates the relationship between obesity and cataract [49], while Mendelian randomization analyses have produced differing conclusions regarding the causal effect of obesity on age-related cataract [52,53].

Potential biological mechanisms include altered glucose metabolism, oxidative stress, and accumulation of sorbitol within the lens [54]. However, the human evidence does not uniformly support a direct causal effect of obesity.

Intervention studies provide additional, although still incomplete, evidence. Bariatric surgery has been associated with lower cataract incidence [55], and treatment with glucagon-like peptide-1 receptor agonists (GLP-1RAs) has been associated with cataract outcomes in observational studies [56,57,58]. These findings require cautious interpretation because treatment selection, diabetes severity, weight loss, and other metabolic factors may influence the observed associations.

Overall, obesity may be associated with increased cataract risk, particularly for age-related and posterior subcapsular cataract, but metabolic factors—especially diabetes—appear to play an important role in this relationship.

## 8. Age-Related Macular Degeneration (AMD)

Obesity and central adiposity have been associated with age-related macular degeneration (AMD), although the strength of the association varies between anthropometric measures and disease stages. Waist circumference and other measures of central adiposity may provide more informative associations than BMI alone [59,60,61,62].

Nutritional factors may contribute to this relationship. Among individuals with obesity, lower intakes of fiber, calcium, phosphorus, potassium, thiamine, niacin, vitamin C, protein, vitamin A, and β-carotene have been associated with AMD in sex-specific analyses [63]. These findings suggest that dietary quality may interact with adiposity, although cross-sectional nutritional data cannot establish whether dietary insufficiency precedes AMD or results from broader differences in health behavior.

Visceral adipose tissue activity has also been associated with AMD severity and systemic inflammation [64], while higher systemic inflammatory response index levels have been associated with AMD risk [65]. Experimental studies provide mechanistic support. High-fat/high-sugar diets can induce retinal lesions, and complement activation, vascular alterations, and RPE abnormalities in experimental models [15,16]. Obesity-related IL-1β signaling may contribute to iron accumulation in the RPE, oxidative stress, and retinal dysfunction [17]. Other experimental work has demonstrated increased oxidative stress and complement activation in the retina under obesity-related metabolic conditions [14].

Nutritional interventions may modify some of these pathways. Lutein and zeaxanthin supplementation can influence retinal lipid metabolism and inflammatory and oxidative-stress pathways in experimental obesity models [66]. Observational studies have also reported lower AMD incidence among patients receiving GLP-1RAs [67,68], although these findings require confirmation in prospective studies.

Overall, obesity may influence AMD through a combination of systemic inflammation, visceral adipose tissue activity, oxidative stress, lipid dysregulation, complement activation, and altered carotenoid availability. Nevertheless, the human evidence remains primarily observational, and the independent contribution of obesity relative to age, diet, smoking, metabolic disease, and other established AMD risk factors remains uncertain.

## 9. Glaucoma

The relationship between obesity and glaucoma is particularly complex because obesity may influence IOP without necessarily increasing susceptibility of the optic nerve to glaucomatous damage.

Several studies have associated higher BMI, waist circumference, or other adiposity measures with increased IOP and ocular hypertension [69,70,71,72,73,74]. Obesity may influence IOP through increased episcleral venous pressure, altered aqueous humor outflow, and metabolic pathways involving ANGPTL7 [24,25]. A reduction in BMI following bariatric surgery has also been associated with a sustained reduction in IOP [75].

However, the relationship between obesity and glaucoma incidence is less consistent. Some studies report increased risk of primary open-angle glaucoma (POAG), particularly in younger individuals or specific sex groups [71,72], whereas others have found inverse or neutral associations [18,76,77]. For example, one study reported a 7% reduction in open-angle glaucoma risk for each one-unit increase in BMI [76], while another found an 11.8% lower risk among individuals with obesity [77].

These apparently divergent findings may reflect the distinction between determinants of IOP and determinants of optic nerve susceptibility. Elevated IOP is an important risk factor for glaucoma, but IOP alone does not determine whether retinal ganglion cells and the optic nerve undergo glaucomatous damage. Vascular dysregulation, neuroinflammation, metabolic dysfunction, and individual susceptibility may modify this relationship.

Metabolic health appears particularly relevant. In the NIH All of Us Research Program, obesity and central obesity were associated with lower odds of glaucoma, whereas metabolic syndrome and increasing metabolic syndrome severity were associated with higher odds [18]. In another large population study, glaucoma risk increased with the number of metabolic syndrome components, with impaired glucose tolerance showing the strongest association [19]. These findings suggest that metabolic dysfunction may be more informative than BMI alone.

The possibility of obesity-related neuroinflammation also warrants consideration. Obesity-associated gut dysbiosis may promote intestinal barrier dysfunction, endotoxemia, and systemic inflammation, potentially contributing to retinal ganglion cell and optic nerve injury [26]. Conversely, some studies have reported that higher BMI is associated with slower glaucoma progression or a lower prevalence of low-tension glaucoma [78,79].

Sex, age, diabetes, blood pressure, lipid status, and the distinction between metabolically healthy and metabolically unhealthy obesity may therefore contribute to the heterogeneous findings. Overall, obesity appears to have a more consistent relationship with elevated IOP than with glaucoma itself. Future studies should distinguish IOP-related effects from IOP-independent optic nerve vulnerability and should incorporate detailed metabolic, vascular, and inflammatory phenotyping.

## 10. Diabetic Retinopathy (DR)

The relationship between obesity and diabetic retinopathy (DR) is strongly influenced by the presence and duration of diabetes. Multiple anthropometric measures—including BMI, body roundness index, conicity index, waist-to-height ratio, tri-ponderal mass index, weight-adjusted waist index, and measures of visceral adiposity—have been associated with DR [80,81,82].

Abdominal obesity appears particularly relevant. A systematic review and meta-analysis found an association between abdominal obesity and DR among individuals with diabetes, although the strength of this association varied between populations and did not consistently correspond to DR severity [81].

The biological relationship is likely multifactorial. Obesity promotes insulin resistance and dyslipidemia and frequently coexists with hypertension and chronic low-grade inflammation. In individuals with diabetes, these factors may act synergistically with chronic hyperglycemia to promote oxidative stress, endothelial dysfunction, and retinal microvascular damage.

The distinction between obesity and diabetes is therefore crucial. Obesity may increase the likelihood of developing diabetes while simultaneously showing different associations with DR after diabetes has developed. For example, one Asian population study reported that overweight and obesity were associated with a higher risk of developing diabetes but a lower subsequent risk of DR [83]. This apparent paradox illustrates the difficulty of interpreting BMI as an independent risk factor without considering diabetes duration, glycemic control, treatment, and survivor or selection effects.

Interventional evidence provides some support for the importance of metabolic improvement. Patients with type 2 diabetes and obesity who underwent Roux-en-Y gastric bypass showed a tendency toward less DR progression, particularly when surgery resulted in diabetes remission [84]. However, these data do not demonstrate that weight reduction alone prevents DR.

Overall, obesity appears to be relevant to DR primarily within a broader metabolic network involving diabetes, hyperglycemia, hypertension, dyslipidemia, insulin resistance, and inflammation. Future studies should therefore assess obesity together with these factors rather than treating BMI as an isolated exposure.

## 11. Retinal Vein Occlusion (RVO)

Retinal vein occlusion (RVO) is a multifactorial retinal vascular disorder in which hypertension, diabetes, dyslipidemia, vascular dysfunction, and inflammatory mechanisms may interact. Oxidative stress is considered an important contributor to retinal vascular injury and disease progression [85].

Recent evidence suggests that visceral adipose tissue activity may also be relevant. Higher metabolic activity of visceral adipose tissue has been associated with RVO and systemic inflammation, suggesting that metabolically active visceral fat may be more informative than BMI alone [86].

However, the relationship between BMI and RVO is inconsistent. In a Korean nationwide cohort, the association between obesity and RVO differed according to diabetes status: among individuals with diabetes, lower BMI was associated with higher RVO risk, whereas among individuals without diabetes, higher BMI was associated with increased risk [87]. Such findings illustrate the potential for effect modification by metabolic status.

Other studies have associated BMI, fat-free mass, fasting glucose, and post-load glucose levels with RVO [88], while hypertension, dyslipidemia, and BMI have been identified as risk factors for branch retinal vein occlusion, including among younger patients [89,90].

In contrast, MR analyses have not consistently supported a direct causal relationship between obesity-related traits and RVO [91,92]. This discrepancy between observational and genetic evidence is important. Observational associations may partly reflect the effects of hypertension, diabetes, dyslipidemia, sedentary behavior, sleep apnea, or other correlated metabolic factors rather than a direct effect of adiposity.

OSA may represent an additional pathway because central and upper-body adiposity increases the likelihood and severity of OSA, which has been associated with RVO [93]. Nutritional deficiencies following bariatric surgery have also been reported in association with CRVO in case reports, although such observations cannot establish causality [94].

Thus, RVO should be regarded as a vascular outcome potentially influenced by a cluster of metabolic, inflammatory, and vascular risk factors rather than as a direct consequence of obesity. The lack of consistent causal evidence emphasizes the need for prospective studies incorporating detailed metabolic phenotyping.

## 12. Uveal Melanoma

Evidence regarding obesity and uveal melanoma is limited and more heterogeneous than for several metabolic or vascular ocular diseases.

Among postmenopausal women, overweight and obesity have been associated with approximately twofold higher odds of choroidal nevus, a potential precursor lesion for uveal melanoma [95]. Hormonal mechanisms, including cumulative exposure to unopposed estrogen, have been proposed as one possible explanation.

Paradoxically, higher BMI has also been associated with lower metastatic risk among patients with uveal melanoma, while higher circulating leptin levels have been associated with more favorable survival characteristics [96]. Alterations in the melanocortin pathway have additionally been proposed, although the functional significance of these observations remains uncertain [97]. Obesity and diabetes have also been associated with earlier-stage presentation in patients with posterior uveal melanoma [98].

These findings should be interpreted cautiously because they are based on relatively limited observational evidence and may reflect complex interactions among metabolic status, hormonal signaling, tumor biology, and immune responses. At present, there is insufficient evidence to conclude that obesity directly influences uveal melanoma development or progression.

## 13. Nutritional Factors and Potential Therapeutic Strategies

### 13.1. Dietary Patterns

A high-fat and high-sugar dietary pattern may promote obesity-related metabolic dysfunction, oxidative stress, and inflammation. Experimental models have demonstrated retinal changes resembling features of AMD following high-fat/high-sugar or “fast-food” dietary exposure [15,16]. These findings provide biological plausibility for the hypothesis that dietary quality may influence ocular health independently of body weight.

However, experimental dietary exposure should not be equated with evidence that a specific human dietary pattern causes ocular disease. Human nutritional studies are often affected by measurement error, dietary clustering, reverse causation, and residual confounding.

### 13.2. Fatty Acids and Lipid Metabolism

Altered lipid metabolism is relevant to both systemic obesity and ocular tissues. In the ocular surface, dyslipidemia and changes in meibum composition may contribute to MGD [20,21,22]. In the retina, altered lipid metabolism may affect carotenoid transport and retinal homeostasis [23].

The biological effects of dietary fat may therefore depend on fatty acid composition rather than total fat intake alone. Further prospective research is needed to determine whether replacing saturated fats with unsaturated fatty acids can modify obesity-related ocular risk.

### 13.3. Antioxidant Nutrients and Carotenoids

Lutein and zeaxanthin are particularly relevant to AMD because they accumulate in the macula and participate in antioxidant and light-filtering functions. Experimental studies indicate that lutein and zeaxanthin isomers may influence oxidative stress, lipid metabolism, and inflammatory signaling in obesity-related retinal dysfunction [66].

Observational evidence also suggests that obesity may be associated with lower macular pigment optical density, potentially reflecting altered carotenoid distribution or competition between adipose tissue and retinal tissue for carotenoid uptake [23]. However, these observations do not establish that supplementation prevents AMD in individuals with obesity.

### 13.4. Dietary Micronutrients

Nutritional inadequacy may be relevant in obesity. In a Korean population study, several nutrient intakes differed between individuals with and without AMD among those with obesity [63]. These findings support the need to consider dietary quality rather than focusing exclusively on caloric intake or BMI.

### 13.5. Weight-Loss Interventions

Weight reduction represents a potentially modifiable exposure. Bariatric surgery has been associated with lower cataract risk [55], reductions in IOP [75], and less pronounced DR progression in some populations [84]. However, bariatric surgery may also introduce nutritional deficiencies, and isolated case reports have described retinal vascular complications after surgery [94].

Pharmacological treatment with GLP-1RAs has also been associated with ocular outcomes, including lower observed rates of AMD and cataract in some studies [56,57,58,67,68]. These findings should be considered preliminary because treatment indication, diabetes status, weight loss, and other metabolic differences may confound associations.

Overall, nutritional and weight-loss interventions are promising but should not yet be interpreted as established ophthalmic preventive therapies. Randomized trials and prospective studies with standardized ophthalmic endpoints are needed.

## 14. Cross-Disease Synthesis

The evidence reviewed here suggests that obesity is unlikely to influence ocular disease through a single pathway. Instead, several overlapping mechanisms may converge on different ocular tissues.

The ocular surface appears particularly sensitive to lipid abnormalities, inflammation, and meibomian gland dysfunction. The cornea may be affected through inflammatory and oxidative mechanisms, while retinal diseases are influenced by metabolic, vascular, inflammatory, and mitochondrial pathways. The optic nerve may represent a distinct situation in which systemic obesity can increase IOP while simultaneously producing metabolic or vascular effects that modify optic nerve susceptibility.

A recurring theme is that central and metabolically active adiposity may be more informative than BMI alone. BMI is a useful population-level measure but does not distinguish visceral from subcutaneous adipose tissue, lean mass from fat mass, or metabolically healthy from metabolically unhealthy obesity. Studies using waist circumference, waist-to-hip ratio, body fat percentage, visceral adiposity indices, or body roundness measures may therefore identify associations that are not apparent when BMI is used alone.

Another recurring issue is confounding by metabolic comorbidities. Diabetes, hypertension, dyslipidemia, OSA, physical inactivity, and dietary quality are all correlated with obesity and may independently influence ocular health. Consequently, an observed association between obesity and an ocular disease does not necessarily indicate that adiposity itself is responsible for the association.

The evidence is also not equivalent across study designs. Observational studies are useful for identifying epidemiological patterns but cannot establish causality. MR studies can provide complementary evidence regarding potential causal relationships but depend on the validity of genetic instruments and assumptions underlying the analysis. Experimental studies provide mechanistic plausibility but may not reproduce the complexity of human obesity. Interventional studies offer the most direct opportunity to determine whether modifying obesity or its metabolic consequences alters ocular outcomes, but such evidence remains limited.

The divergent results observed for glaucoma, myopia, cataract, and RVO therefore do not necessarily indicate that the literature is contradictory. Instead, they may reflect differences in disease biology, population characteristics, adiposity measures, metabolic status, environmental exposures, and study design.

## 15. Limitations and Future Research

Several limitations of the available evidence should be acknowledged.

First, most clinical studies examining obesity and ocular disease are cross-sectional or observational. These designs can identify associations but cannot establish a direct causal relationship and remain vulnerable to confounding and reverse causation.

Second, the definition and measurement of obesity vary substantially across studies. BMI, waist circumference, waist-to-hip ratio, body fat percentage, and visceral adiposity indices reflect different dimensions of adiposity and should not be considered interchangeable. This methodological heterogeneity limits direct comparisons between studies and may contribute to inconsistent findings.

Third, this narrative review did not include a formal quality assessment or risk-of-bias evaluation. Consequently, the overall strength of the evidence should be interpreted cautiously because the included studies differ in design, sample size, methodology, and potential sources of bias. Future systematic reviews and meta-analyses incorporating standardized quality appraisal are warranted.

Fourth, many studies do not adequately distinguish obesity from its associated metabolic conditions. Diabetes, hypertension, dyslipidemia, OSA, physical inactivity, and dietary factors may act as confounders, mediators, or effect modifiers. Future research should therefore use multidimensional metabolic phenotyping rather than relying exclusively on BMI.

Future prospective cohorts should combine anthropometric measurements with body-composition analysis, metabolic biomarkers, inflammatory markers, dietary assessments, physical-activity data, and standardized ophthalmic phenotyping. Longitudinal studies should also investigate whether changes in body composition and metabolic health precede changes in ocular structure or function.

Interventional research is particularly important. Studies of dietary modification, physical activity, pharmacological weight loss, bariatric surgery, and metabolic disease treatment should include predefined ocular endpoints where appropriate. Such research could determine whether associations observed in epidemiological studies are modifiable and whether treatment of obesity has ophthalmic benefits independent of improvements in diabetes, hypertension, or lipid metabolism.

## 16. Conclusions

Obesity and obesity-related metabolic dysfunction are associated with a broad spectrum of ocular conditions, including dry eye disease, keratoconus, myopia, cataract, age-related macular degeneration, glaucoma, diabetic retinopathy, retinal vein occlusion, and potentially uveal melanoma. However, the strength and direction of these associations vary considerably across diseases and populations.

Across the available literature, chronic low-grade inflammation, oxidative stress, mitochondrial dysfunction, insulin resistance, dyslipidemia, endothelial dysfunction, adipokine imbalance, and immune dysregulation emerge as interconnected mechanisms that may influence ocular tissues. These pathways provide a plausible biological framework linking systemic metabolic dysfunction with ocular pathology, but mechanistic plausibility should not be interpreted as evidence of causality.

The heterogeneous findings also demonstrate that obesity should not be treated as a uniform exposure. BMI, central adiposity, visceral fat, body composition, metabolic health, dietary quality, and obesity-related comorbidities may have distinct relationships with ocular outcomes. The particularly inconsistent evidence for glaucoma and retinal vein occlusion illustrates the importance of distinguishing obesity itself from the metabolic and vascular conditions that frequently accompany it.

From a nutritional perspective, dietary quality, fatty acid composition, antioxidant status, and carotenoid availability may modify some obesity-related ocular pathways. Experimental studies provide evidence that high-fat/high-sugar diets can promote retinal oxidative stress, inflammation, complement activation, and structural abnormalities, whereas selected nutritional and weight-loss interventions show potential benefits. Nevertheless, clinical evidence remains insufficient to establish specific dietary interventions as preventive treatments for obesity-related ocular disease.

Clinically, these findings support closer integration of ophthalmic, metabolic, and nutritional assessment, particularly in individuals with central obesity, diabetes, hypertension, dyslipidemia, or other features of metabolic dysfunction. Rather than considering BMI alone, clinicians and researchers should increasingly focus on the broader metabolic phenotype.

Future research should prioritize prospective longitudinal studies, standardized definitions of adiposity, integration of nutritional and metabolic data, mechanistic investigations, and well-designed intervention trials. Such approaches are necessary to distinguish direct effects of adiposity from pathways mediated by diabetes, vascular dysfunction, inflammation, lifestyle, and other confounding factors and may ultimately facilitate more precise prevention and management of obesity-associated ocular disease.

## Figures and Tables

**Table 1:** Common mechanisms linking obesity to ocular pathology. The upward arrow means an increase.

Mechanism	Obesity-Related Alteration	Obesity Consequence	Diseases Potentially Affected
Chronic inflammation	↑ TNF-α, IL-6, IL-1β	Tissue inflammation and immune activation	DED, keratoconus, AMD, glaucoma, DR, RVO
Oxidative stress	↑ ROS, impaired antioxidant defense	Cellular and mitochondrial injury	Keratoconus, cataract, AMD, DR, RVO
Mitochondrial Dysfunction	Altered energy metabolism	Retinal and RPE dysfunction	AMD, DR, neurodegenerative diseases
Insulin Resistance	Impaired glucose utilization	Hyperglycemia, AGE/RAGE signaling	Cataract, DR, glaucoma
Dyslipidemia/lipotoxicity	↑ TG, altered cholesterol metabolism	Meibum dysfunction, retinal lipid accumulation, vascular injury	DED/MGD, AMD, RVO
Endothelial Dysfunction	Vascular inflammation and impaired perfusion	Retinal microvascular dysfunction	AMD, DR, RVO, glaucoma
Adipokine Dysregulation	Altered leptin/other adipokines	Inflammation and vascular signaling	AMD, glaucoma, retinal diseases
Gut Dysbiosis	Barrier dysfunction/endotoxemia	Systemic and neuroinflammation	Glaucoma, retinal diseases
ANGPTL7 Pathway	Increased ANGPTL7	Increased resistance to aqueous outflow/IOP	Glaucoma
Nutrient/Carotenoid Dysregulation	Altered lutein/zeaxanthin availability	Reduced macular antioxidant protection	AMD

## Data Availability

All the materials and information will be available upon an e-mail request to the corresponding author.
